# Push-Pull Receptive Field Organization and Synaptic Depression: Mechanisms for Reliably Encoding Naturalistic Stimuli in V1

**DOI:** 10.3389/fncir.2016.00037

**Published:** 2016-05-11

**Authors:** Jens Kremkow, Laurent U. Perrinet, Cyril Monier, Jose-Manuel Alonso, Ad Aertsen, Yves Frégnac, Guillaume S. Masson

**Affiliations:** ^1^Institut de Neurosciences de la Timone, UMR 7289, Centre National de la Recherche Scientifique - Aix-Marseille UniversitéMarseille, France; ^2^Neurobiology and Biophysics, Faculty of Biology, University of FreiburgFreiburg, Germany; ^3^Bernstein Center Freiburg, University of FreiburgFreiburg, Germany; ^4^Department of Biological Sciences, State University of New York (SUNY-Optometry)New York, NY, USA; ^5^Unité de Neurosciences, Information et Complexité, UPR Centre National de la Recherche Scientifique 3293Gif-sur-Yvette, France

**Keywords:** natural visual stimuli, visual cortex, push-pull receptive field, excitation/inhibition, sensory coding

## Abstract

Neurons in the primary visual cortex are known for responding vigorously but with high variability to classical stimuli such as drifting bars or gratings. By contrast, natural scenes are encoded more efficiently by sparse and temporal precise spiking responses. We used a conductance-based model of the visual system in higher mammals to investigate how two specific features of the thalamo-cortical pathway, namely push-pull receptive field organization and fast synaptic depression, can contribute to this contextual reshaping of V1 responses. By comparing cortical dynamics evoked respectively by natural vs. artificial stimuli in a comprehensive parametric space analysis, we demonstrate that the reliability and sparseness of the spiking responses during natural vision is not a mere consequence of the increased bandwidth in the sensory input spectrum. Rather, it results from the combined impacts of fast synaptic depression and push-pull inhibition, the later acting for natural scenes as a form of “effective” feed-forward inhibition as demonstrated in other sensory systems. Thus, the combination of feedforward-like inhibition with fast thalamo-cortical synaptic depression by simple cells receiving a direct structured input from thalamus composes a generic computational mechanism for generating a sparse and reliable encoding of natural sensory events.

## Introduction

**Figure 1:**
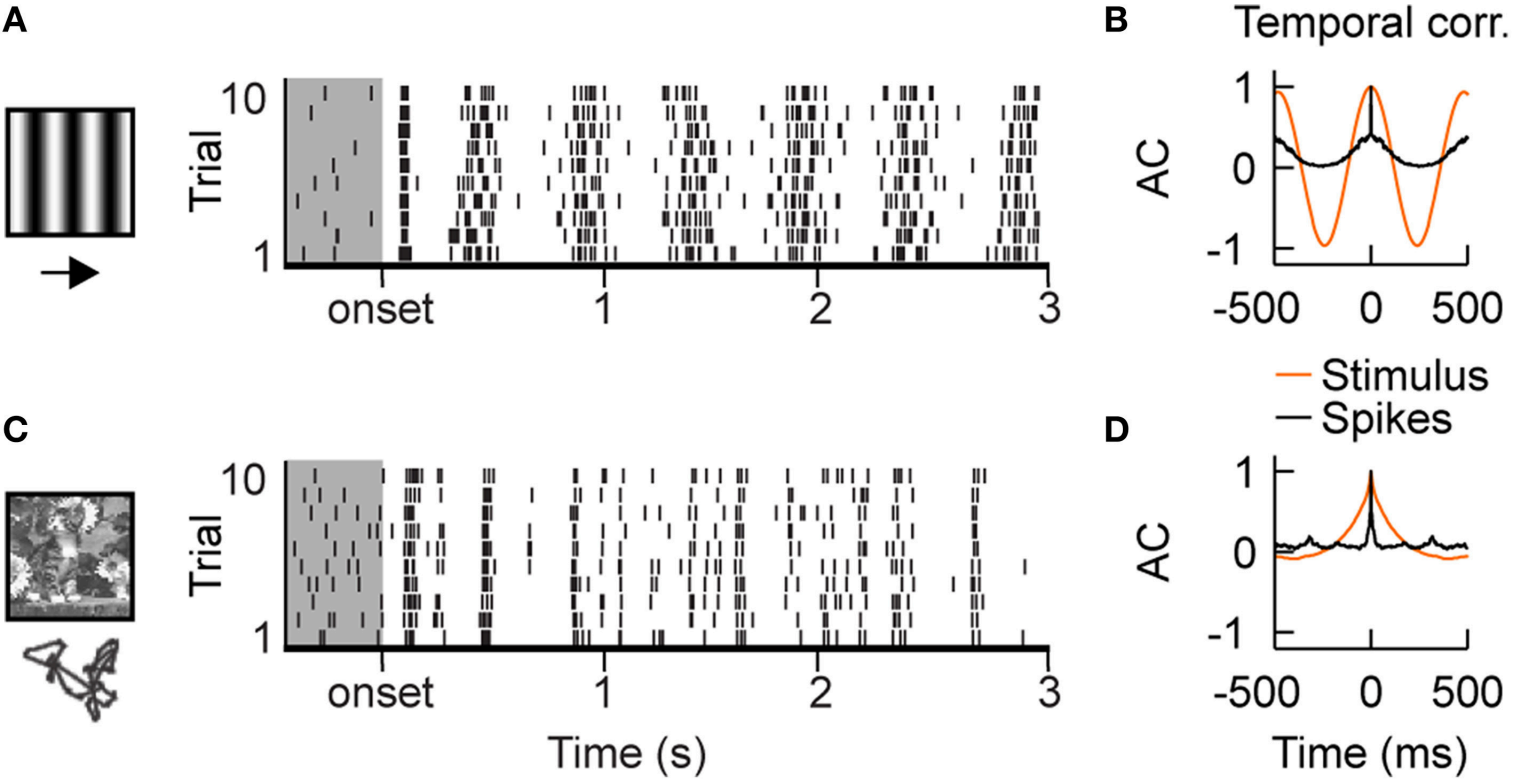
**Intracellular recordings of V1 simple cells *in vivo*. (A)** Stimulus driven spiking responses of simple cells in the primary visual cortex (V1) of cat are dense and variable during the presentation of drifting gratings, shown by the raster plot. Gray shaded region shows ongoing activity. **(B)** Temporal auto-correlation functions: the average spiking response (black) follows the temporal frequency of the grating stimulus (orange). **(C)** Superimposing eye-movements on a natural image results in sparse responses with diverse trial-to-trial spike time variability. Both, temporally precise and imprecise spiking responses can be observed. **(D)** Temporal auto-correlation: During natural stimulation the temporal correlation of the average spiking response (black) is shorter than the temporal correlations in the stimulus contrast dynamics (orange). The *in vivo* recordings in V1 have been conducted in the group of Yves Frégnac (Baudot et al., 2013).

Simple cells, in the thalamic recipient layers of area V1, exhibit spatial segregation and contrast opponency between their spiking ON- and OFF-subfields (Hirsch et al., 1998; Martinez et al., 2005). As a consequence, their spiking follows the driving temporal frequency of drifting gratings of optimal orientation and spatial frequency (Figures 1A,B). However, the firing is dense and the exact spike timing of the evoked discharge is highly variable from one trial to another (Tomko and Crapper, 1974; Tolhurst et al., 1983; Baudot et al., 2013). By contrast, the same neurons exhibit sparse and reliable spiking activities when simulated with natural visual stimuli (Figure 1C; Vinje and Gallant, 2000, 2002; Haider et al., 2010; Herikstad et al., 2011; Baudot et al., 2013). Moreover, in contrast with gratings, the timescale of the neuronal responses becomes shorter than the time constant of the autocorrelation function of the natural stimulus contrast dynamics (Figure 1D). It is still not completely understood what mechanisms underlie these differences between artificial and naturalistic conditions.

Cortical inhibition is one potential candidate as the balance and temporal interplay between excitation and inhibition are key factors in determining spiking pattern precision in neuronal networks (Gerstein and Mandelbrot, 1964; Wehr and Zador, 2003; Kumar et al., 2008a; Vogels and Abbott, 2009; Kremkow et al., 2010a,b; Renart et al., 2010; Baudot et al., 2013; Graupner and Reyes, 2013). Furthermore, in sensory cortical areas inhibition is stimulus dependent and has been linked to a diversity of roles in sensory processing (Anderson et al., 2000; Hirsch et al., 2003; Monier et al., 2003; Wehr and Zador, 2003; Priebe and Ferster, 2005; Wilent and Contreras, 2005; Okun and Lampl, 2008; Haider et al., 2010; Liu et al., 2011; Tan et al., 2011; Baudot et al., 2013; Xue et al., 2014; Li et al., 2015a). For example, there is a diversity of excitatory/inhibitory tuning properties in V1 neurons (Monier et al., 2003, 2008; Cardin et al., 2010; Baudot et al., 2013) specially when sampled across all cortical layers (Baudot et al., 2013). Several studies have shown that, in simple cells of higher mammals drifting gratings at preferred orientation cause anti-correlated/out-of-phase excitation and inhibition at the driving frequency (Anderson et al., 2000; Monier et al., 2003; Priebe and Ferster, 2005; Baudot et al., 2013). In contrast, gratings of non-preferred orientation and natural stimuli induce a more complex interplay between excitation and inhibition, with excitation and inhibition being correlated during natural stimuli (Haider et al., 2010; Baudot et al., 2013). Thus, the same cortical cell might exhibit various firing regimes in response to different stimulus statistics, which impose dynamic changes in the balance state and/or the relative timing between excitatory and inhibitory inputs. However, the mechanisms of this stimulus dependent re-shaping of excitation/inhibition are still not fully understood.

Both feedforward and feedback processing could contribute to this contextual modulation of excitation and inhibition in V1 neurons. Center-surround interactions, which likely originate from feedback pathways or horizontal cortical projections (Angelucci et al., 2002; Chavane et al., 2011), are known to modulate neuronal responses during both artificial and natural stimuli (Angelucci et al., 2002; Seriès et al., 2003; Guo et al., 2005; Haider et al., 2010; Nortmann et al., 2015), including changes in the balance of excitation and inhibition (Haider et al., 2010). Thus, recurrent cortical processing is one element that plays an important role in sensory processing during natural viewing (Vinje and Gallant, 2000, 2002; Haider et al., 2010; Onat et al., 2011).

Likewise, the architecture of the thalamo-cortical visual system contains circuit elements that are well suited to modulate excitation and inhibition along the feedforward pathway in a stimulus dependent manner: the push-pull receptive field organization of V1 simple cells (Palmer and Davis, 1981; Ferster, 1988; Tolhurst and Dean, 1990; Hirsch and Martinez, 2006). Here afferent projections from ON-center and OFF-center cells of the visual thalamus (lateral geniculate nucleus “LGN”) provide direct excitatory and indirect di-synaptic inhibitory inputs to simple cells in layer 4 of V1 (Hirsch et al., 1998; Troyer et al., 1998; Martinez et al., 2005; Hirsch and Martinez, 2006). Importantly the ON/OFF receptive fields of simple cells in V1 are organized in an antagonistic “push-pull” manner (Martinez et al., 2005), i.e., flashing a light square on the ON subfield causes excitation while flashing a dark square at the same location causes inhibition (Hirsch et al., 1998). Thus, stimulus dependent interactions of excitation and inhibition occur already within the classical receptive field of simple cells. Please note, while the majority of simple cells express this antagonistic behavior, a small fraction of simple cells also shows push-null or push-push behavior (Martinez et al., 2005) and V1 neurons can show an overlap between excitatory and inhibitory receptive subfields (Cardin et al., 2010) specially outside layer 4.

A classical model for the push-pull receptive field organization of simple cells suggests that the pull/inhibition originates from cortical inhibitory neurons having receptive fields with opposite contrast polarity (ON/OFF) as the target cell (Troyer et al., 1998; Lauritzen et al., 2001; Miller et al., 2001; Lauritzen and Miller, 2003). This model can explain why drifting gratings at preferred orientation cause anti-correlated excitation and inhibition in V1 simple cells (Anderson et al., 2000; Monier et al., 2003; Priebe and Ferster, 2005; Tan et al., 2011; Baudot et al., 2013). However, how the push-pull receptive field organization of simple cells operates under natural viewing conditions, and thus contributes to the contextual reshaping of V1 responses, is unknown.

In addition to cortical inhibition, short-term synaptic dynamics is another potential candidate to shape sensory processing in a contextual manner. For example, short-term synaptic depression of excitatory synaptic transmission (Abbott et al., 1997; Markram et al., 1998) may dynamically regulate feedforward transmission from the LGN to their target simple cells in V1 (Gil et al., 1997; Swadlow and Gusev, 2001; Castro-Alamancos and Oldford, 2002; Freeman et al., 2002; Banitt et al., 2007; Reinhold et al., 2015). Functionally, synaptic depression was suggested to be involved in contrast invariant orientation tuning (Banitt et al., 2007), gain control (Abbott et al., 1997; Rothman et al., 2009), redundancy reduction (Goldman et al., 2002) and it was shown to promote transient discharges in evoked responses (Chance et al., 1998). Thus, short-term synaptic depression at the thalamo-cortical synapse could contribute to stimulus dependent responses in V1. However, it has been argued that short-term depression may not play a prominent role in sensory processing under *in vivo* conditions as here the ongoing activity maintains synapses in a steady level of depression (Boudreau and Ferster, 2005). In contrast to this argument, recordings in the somatosensory system have clearly shown that synaptic depression at the thalamo-cortical synapse does contribute to sensory evoked cortical responses under *in vivo* conditions (Swadlow and Gusev, 2001; Chung et al., 2002; Reinhold et al., 2015). Furthermore, natural visual stimuli cause non-stationary neuronal responses at the level of the LGN *in vivo* with epochs of high activity being interleaved with quiet periods (Butts et al., 2007, 2010; Desbordes et al., 2008). The average interspike interval between these active events is ~ 100–200 ms (Butts et al., 2010) which is enough time for synapse recovery. Therefore, short-term depression at the LGN-V1 synapse could shape cortical responses in a stimulus dependent manner, however, the functional impact on natural scene encoding in V1 has not been studied so far.

In summary, push-pull receptive field organization and thalamo-cortical feedforward depression are two prominent mechanisms contributing to the sensory processing in the thalamo-cortical visual system. However, the functional role of these two respective mechanisms in the processing of artificial and naturalistic visual inputs is still not fully understood. The main focus of this study is to study a conductance-based model of the thalamo-cortical visual pathway implementing the push-pull receptive field organization of V1 simple cells (Troyer et al., 1998) during artificial and natural stimuli. Our reasoning is to have a detailed modeling of this elementary circuit, yet to minimize its complexity by only implementing a limited number of neurons, in order to be able to analyze the whole range of possible dynamical states. In particular, to illustrate the biological relevance of our modeling results, we compare the results of our simulations with *in vivo* responses from cat V1 (group of YF and published in Baudot et al., 2013) and LGN (group of JMA) to the same drifting gratings and natural movies as used in our computational study.

## Materials and methods

### *In vivo* recordings

All electrophysiological recordings were conducted in the anesthetized and paralyzed cat. The group of Yves Frégnac conducted the intracellular recordings of V1 neurons with the methods previously described (Fournier et al., 2011; Baudot et al., 2013). All surgical procedures and animal experimentation were performed in conformity with national (JO 87-848) and European (86/609/CEE) legislations on animal experimentation, and strictly following the recommendations of the Physiological Society, the European Commission and NIH. The extracellular spiking activity of LGN neurons was recorded in the lab of Jose-Manuel Alonso using methods described in Jin et al. (2011), Kremkow et al. (2014). All procedures were performed in accordance to the guidelines of the US Department of Agriculture and approved by the Institutional Animal Care and Use Committee at the State University of New York, State College of Optometry.

### Stimuli

Stimuli were modeled as animated sequences of images (**Figure 3A**) presented at a refresh rate of 150 Hz, similar to the refresh rate of the monitors used in the *in vivo* experiments. Two types of stimulus conditions are compared here: (1) drifting grating and (2) naturalistic movies. The drifting sinusoidal grating had a spatial frequency of 0.8 cpd and a temporal frequency of 2 Hz. We generated a realistic, natural movie by shifting a natural image (a cat in a flower field) according to an eye movement scan path. The eye movement scan path was generated by the same model of naturally occurring eye-movements as used in the intracellular experiments in V1 (Baudot et al., 2013). Note that, although the exact same pattern of eye-movements was replicated here in the model and *in vivo*, stimulation variations may still remain due to differences in the initial fixation position in the image, the receptive field size, shape and to differences in the resolution (pixel/degree) of the image.

### Models

We built a conductance-based model of the thalamo-cortical pathway including integrate-and-fire neuron models of LGN and V1 simple cells to investigate different aspects of neural coding in V1 simple cells (**Figure 3**). The conductance based nature of the model allowed us to investigate the effect of both cortical inhibition and feedforward depression on the neuronal coding of V1 simple cells during natural stimuli and their impact on sub-threshold membrane potential Vm dynamics. Spiking input to V1 was obtained from two populations of ON-center and OFF-center geniculate neurons by convolving the stimuli with the linear receptive fields of the LGN neurons followed by a non-linear spiking mechanism (**Figures 3A,B**). Feed-forward depression at the thalamic input onto cortical cells was modeled by short-term plasticity (Abbott et al., 1997; Chance et al., 1998; Markram et al., 1998; Tsodyks et al., 2000; Banitt et al., 2007). The V1 model corresponded to a prototypical push-pull network of reciprocally connected excitatory and inhibitory neurons in the thalamic input layers (Troyer et al., 1998; **Figure 3C**). In this framework, excitatory and inhibitory neurons are more likely to be connected to neurons with similar orientation preference (**Figure 3C**, right). Excitatory connections are established between neurons of similar phase but inhibitory neurons connect preferentially to cells having opposite phase. Such connectivity effectively implements the push-pull receptive field organization of V1 simple cells (Palmer and Davis, 1981; Ferster, 1988; Tolhurst and Dean, 1990; Hirsch and Martinez, 2006). We now provide the details of both neurons and networks.

#### Neurons

Neurons in the LGN and V1 were modeled as leaky-integrate-and-fire neurons, with the sub-threshold dynamics of the membrane potential in neuron i described by the following equation:

(1) $$C\frac{d}{dt}V^{i}\left(t\right)+G_{rest}\left[V^{i}\left(t\right)-V_{rest}\right]=I_{syn}^{i}$$

Where $I_{syn}^{i}$ is the total synaptic input current into neuron *i*, and *C* and *G*_*rest*_ denote the passive electrical properties of its membrane at rest (*V*_*rest*_). When the membrane potential reaches a fixed spike threshold Vth above rest, a spike is emitted, the membrane potential is reset to its resting value, and synaptic integration is halted for 2 ms, mimicking the refractory period in real neurons. The parameters used in the simulations were:

$$\begin{array}{l} \text{Excitatory neurons}:\;C\;=290\;pF,\;G_{rest}\;=\;29\;nS,\\ \;V_{rest}=-70\;mV\;V_{th}=-57\;mV\\ \text{Inhibitory neurons}:\;C\;=141\;pF,\;G_{rest}\;=\;22\;nS,\\ \;V_{rest}=-70\;mV\;V_{th}=-57\;mV \end{array}$$

Synaptic inputs are modeled as transient conductance changes, using exponential functions with τ_*exc*_ = 3 ms and τ_*inh*_ = 10 ms (Kuhn, 2004; Muller et al., 2007; Kumar et al., 2008a; Kremkow et al., 2010b). Excitatory and inhibitory synaptic delays in the V1 network are set to 2 ms. We used the model developed by Tsodyks et al. (2000) to implement short-term synaptic plasticity. Please refer to the Equations (3) and (4) in the original publication (Tsodyks et al., 2000) and to its implementation in the simulation environment NEST (Morrison et al., 2005; Eppler et al., 2008) for further details.

#### Model of the visual thalamus and of the input layer of primary visual cortex

As illustrated in **Figure 3**, the model of the early visual system was composed of a small patch of visual thalamus (LGN) and a small patch of the thalamic input layer of primary visual cortex (V1), both covering the same area of the visual field. The LGN provided feed-forward inputs to V1. Neurons in V1 were recurrently connected but feedback projections from V1 to LGN were not included. The basic structure of the LGN and V1 models was taken from the literature and described in detail below.

#### Visual thalamus: lateral geniculate nucleus (LGN)

We used a standard model, similar to Troyer et al. (1998), in order to construct a realistic dense LGN, covering 6.8 × 6.8° of visual field with a lattice of 61 × 61 ON cells and 61 × 61 OFF cells (**Figure 3B**). Each LGN cell had a characteristic spatiotemporal receptive field, with its spatial center-surround profile defined by a difference of Gaussians and its bi-phasic temporal profile as a difference of Gamma functions (Cai et al., 1997; Troyer et al., 1998). The main parameters of the spatiotemporal receptive field were taken from Allen and Freeman (2006). The size of the center (σ_*center*_) was chosen to match the subfield size of the cortical neurons (Reid and Alonso, 1995), and the surround extent was defined as: σ_*surround*_ = 1.5${}^{*}\sigma_{\text{center}}+$0.4 (Allen and Freeman, 2006). To elicit stimulus-dependent spiking in a given LGN neuron, the spatiotemporal receptive field was convolved with the stimulus (i.e., a sequence of images) and the resulting filtered stimulus gave the generating current (GC) which was injected into the LGN neuron (Pillow et al., 2005) in order to induce stimulus dependent spiking responses (**Figure 3B**). LGN neurons were modeled as leaky integrate-and-fire neurons. The GC was multiplied by a linear scaling factor to map the 0–100% contrast. In addition to the feedforward sensory drive, LGN neurons received white noise current input to introduce trial-by-trial variability. This white noise input was calibrated such that the thalamic neurons elicited a spontaneous uncorrelated spiking activity (at around 10 spikes/s) in the absence of a visual stimulus (Troyer et al., 1998).

#### Visual cortex: thalamo-cortical layer of primary visual cortex (V1)

The network of the thalamo-cortical layer of the primary visual cortex model was adapted from Troyer et al. (1998). It contained 1600 excitatory (E) and 400 inhibitory (I) neurons (ratio 4:1) (Kumar et al., 2008b), simulating a local cortical network. The cortical receptive fields had elongated subfields (**Figure 3C**), described by a Gabor function with an aspect ratio of 3.3 (Jones and Palmer, 1987). The parameters of the Gabor were chosen such that each main subfield width matched the diameter of the center of the LGN receptive field (Reid and Alonso, 1995). The orientation of the Gabor was randomly drawn from a uniform distribution between 0 and 180° and its phase between 0 and 360°. Probabilistic sampling of the Gabor function yield around ~60–100 incoming synapses from the LGN (~30–50 from ON-center cells and ~30–50 from OFF-center cells) and is in the range used in Banitt et al. (2007). Synaptic weights were normalized by the value of the Gabor function and scaled such that each cortical neuron received similar amount of total conductance from LGN (Troyer et al., 1998). This value was chosen such that each individual synapse was weak and below 0.6~mV PSP amplitude at rest (Bruno and Sakmann, 2006). This approach establishes the orientation preferences from the LGN afferents (Ferster et al., 1996). In addition, the thalamo-cortical synapses onto both excitatory and inhibitory neurons showed synaptic depression (**Figure 3B**) with the basic parameters similar to Banitt et al. (2007) (U = 0.3, τ_psc_ = 3 ms, τ_fac_ = 21 ms and τ_rec_ ranging from 1 to 110 ms). The excitatory thalamo-cortical synaptic input to the inhibitory neurons was scaled by a factor 2 to induce effective cortical inhibition (Cruikshank et al., 2007; Kremkow et al., 2010b).

All cortical neurons had central receptive field positions located within ±0.2° of the same visual position, taking into account the cortical magnification factor (Troyer et al., 1998). The optimal spatial frequency was set to 0.8 cpd, similar to the *in vivo* neurons. Due to the small receptive field size of the cortical neurons, some of the LGN neurons did not connect to cortical neurons as they fell outside their classical receptive fields. The cortico-cortical connections were correlation-based (Miller, 1994; Troyer et al., 1998; Lauritzen et al., 2001), such that the probability of two neurons having a connection depends on the orientation and phase difference between their receptive fields (Troyer et al., 1998). For excitatory synapses, the Gaussian connection probability peaks at the same orientation and phase (σ_orientation_ = 15°, σ_phase_ = 30°) (**Figure 3C**). For inhibitory synapses, the connection probability peaked at the same orientation, but with a phase difference of 180°, resulting in an anti-phase, “push-pull” behavior (**Figure 3C**, righ; Troyer et al., 1998). All excitatory synaptic weights were small (~1.5 nS, ~0.1–0.5 mV) (Matsumura et al., 1996; Troyer et al., 1998) such that multiple synchronous inputs were needed to elicit spiking (Bruno and Sakmann, 2006). The synaptic weight of the inhibitory neurons ranged from 0 to 0.9 nS depending on the level of push-pull inhibition (inhibitory gain, see below).

This connectivity scheme results in about ~90 cortico-cortical synapses. We are fully aware that this number underestimates the real number of cortico-cortical synapses. However, our model attempts to represent only the local connectivity in the thalamo-cortical input layer, as synapses from other layers and more distant locations are not included. In fact, it has been recently shown that a considerable fraction (>74%) of excitatory synapses originate from non-local locations (>500 μm radial distance) (Stepanyants et al., 2009; Boucsein et al., 2011). As the model represents only a small local cortical area (a radius of ~200 μm), the number of potential local synapses may even be lower (cf. Figure 1C in Stepanyants et al., 2009, Figure 4 in Boucsein et al., 2011) for distance-dependent fraction of local excitatory neurons). In addition to the structural connectivity described above, the synapses between excitatory neurons in the recurrent network in V1 were modeled with short-term plasticity synapses (Troyer et al., 1998) to mimic depressing synapses (Tsodyks and Markram, 1997; Markram et al., 1998). The cortico-cortical values were taken from Markram et al. (1998), Troyer et al. (1998), Banitt et al. (2007), Haeusler and Maass (2007) (U = 0.5, τ_psc_ = 3 ms, τ_fac_ = 50 ms and τ_rec_ = 1100 ms) and resulted in a strong depression between excitatory neurons that was essential for the stability of the recurrent network (Troyer et al., 1998). For the sake of simplicity, and because the amount of depression and facilitation was less clear for excitatory synapses onto inhibitory neurons, and for inhibitory synapses in general, all these remaining synapses were assumed to be static. The noise level in the cortical neurons was adjusted by providing Poisson distributed spiking background input. This results in excitatory and inhibitory conductance without sensory stimulus from the LGN. The background rate was set such that, together with the ongoing activity in the LGN, membrane potential fluctuations in the cortical neurons were kept in the range (~2–3 mV) observed *in vivo* (Destexhe et al., 2003; Sadagopan and Ferster, 2012; Baudot et al., 2013).

#### Inhibitory gain

To scale the strength of the push-pull inhibition, we changed the peak amplitude of the inhibitory synapses in the V1 network. As mentioned above, excitatory and inhibitory synapses have different time constants (τ_exe_ = 1.5 ms and τ_inh_ = 10 ms) (Kuhn, 2004; Muller et al., 2007). Therefore, we defined the “inhibitory gain” as the ratio between the area of the inhibitory synaptic response (IPSP) and the area of an excitatory synaptic response (EPSP) of amplitude 1 nS, at resting potential. For example, setting the peak inhibitory synaptic conductance to 0.3 nS resulted in an inhibitory gain of 2 at rest.

#### Feedforward depression

To scale the strength of the feedforward depression from the LGN to excitatory and inhibitory neurons in V1, we changed the recovering time constant (τ_rec_) between 1 and 110 ms, with small values resulting in weak depression and larger values in stronger depression (Banitt et al., 2007).

### Data analysis

We performed the following analyses on the spiking activity of neurons. In the model, we selected V1 neurons with preferred orientation similar (±5°) to the grating stimulus.

#### Stimulus evoked firing rates

The mean firing rate of all selected neurons was used as a measure of the stimulus evoked neuronal spiking response.

#### Response timescale

To estimate the time scale of the response and, thereby, the response precision, we used the method originally described in the visual thalamus by Butts et al. (2007). In short, the Peri-Stimulus-Time-Histogram (PSTH) was calculated by binning the spike responses of all trials at 1 ms resolution. The auto-correlation (AC) of the PSTH was then used to characterize the temporal precision of the response. To do so, a Gaussian function was fitted to the auto-correlogram and the resulting σ specifies the temporal precision. A temporally narrow response would result in a small σ, whereas a temporally broad response would yield a large σ.

#### Response reliability

The response reliability was estimated by binning the spike responses of all individual trials at a very high temporal resolution (1 ms), ensuring that maximally one spike could fall within a bin (i.e., binary vector). Calculating the correlation coefficient between the binary vectors of two different trials results in a measure of response reliability (see Aertsen et al., 1979). Repeating this approach for all combinations of trials yielded our measure of response reliability. This measure converges to a value of one for progressively more identical binary vectors, hence when the response is completely reliable.

### Simulation and analysis tools

All network simulations of the conductance-based model were written in python (http://www.python.org) using PyNN (Davison et al., 2008) (http://neuralensemble.org/trac/PyNN) as an interface to the simulation environment NEST (Morrison et al., 2005; Eppler et al., 2008) (http://www.nest-initiative.org). The dynamic equations were integrated at a fixed temporal resolution of 0.1 ms. Simulation management was performed using the python package NeuroTools (https://github.com/NeuralEnsemble/NeuroTools). Data was analyzed in python using the scientific libraries SciPy (http://www.scipy.org) and NumPy (http://www.numpy.org/) and visualized using the plotting library Matplotlib (http://matplotlib.org/) or in Matlab (MathWorks).

## Results

The cell responses illustrated in Figure 1 are taken from a published study conducted by coauthors CM and YF in cat area V1 (Baudot et al., 2013). They clearly illustrate the two different dynamical regimes observed with either drifting gratings or dynamical natural scenes. Our objective was to systematically investigate how push-pull receptive field organization of V1 simple cells and feedforward synaptic depression at the thalamo-cortical synapse could contribute to this contextual reshaping of V1 responses. Therefore, an essential first step was to get a better understanding on how LGN cells respond to these two stimuli, as this would provide important insights into the dynamics of the V1 input under both stimulus conditions. Drifting gratings, other artificial and natural images/movies stimuli have been extensively used to characterize LGN response properties (Ferster et al., 1996; Cai et al., 1997; Kara et al., 2000; Mante et al., 2005, 2008; Allen and Freeman, 2006; Butts et al., 2007, 2010, 2011; Sadagopan and Ferster, 2012). These studies show that LGN cells follow the driving frequency of a drifting grating (Carandini et al., 2005; Mante et al., 2005) and that natural visual stimuli are encoded in an episodic manner, i.e., epochs increased evoked activity are interleaved with quiet epochs (Butts et al., 2010). However, despite this vast amount of literature on LGN responses, a direct comparison of how the same LGN cells respond to a drifting grating and the natural stimulus used in Baudot et al. (2013) (natural image and eye movements) unfortunately did not exist. Therefore, as a first step we recorded extracellular activity of single neurons in cat LGN with the same set of stimuli used in Baudot et al. (2013) to get a better understanding on how these two stimuli are encoded in the LGN *in vivo*. Below, we will first illustrate these LGN responses and then describe the model architecture and explore how synaptic depression and push-pull inhibition along the thalamo-cortical pathway shape sensory processing during artificial and natural inputs.

### LGN activity during drifting gratings and natural stimuli *in vivo*

**Figure 2:**
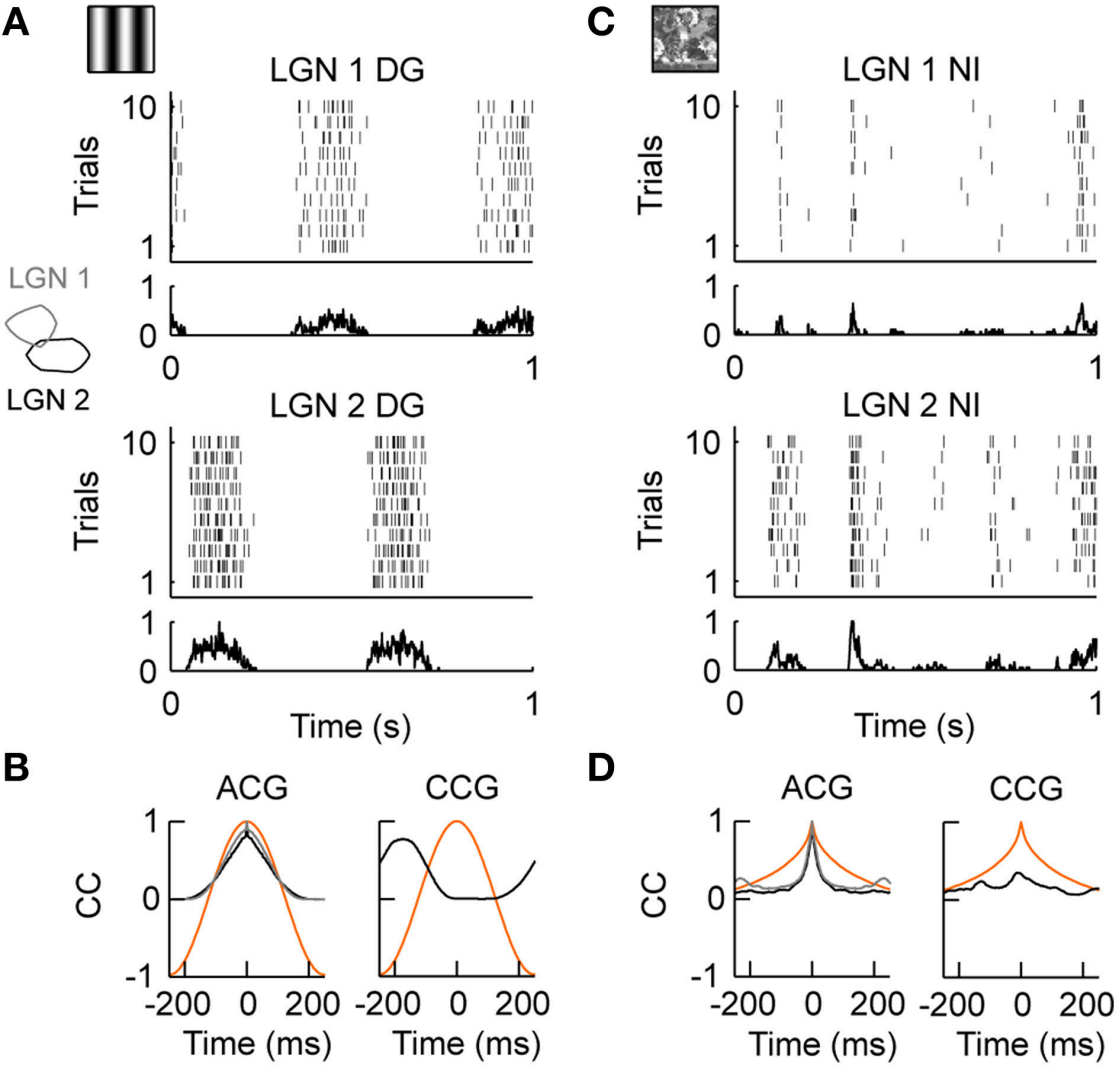
**LGN activity during drifting gratings and natural stimuli *in vivo*. (A)** Neuronal activity in the cat's Lateral Geniculus Nucleus (LGN) during the presentation of drifting gratings. Spiking responses of two simultaneously recorded OFF-center cells (LGN 1, LGN 2) are shown. The dense and variable response at the temporal frequency (TF = 2 Hz) of the grating is evident. The receptive fields of cells LGN 1 and LGN 2 are shown as outlines in the inset on the left. **(B)** Left, temporal auto-correlation (ACG): the average spiking response (gray = LGN 1, black = LGN 2) follows the temporal frequency of the grating stimulus (orange). Right, temporal cross-correlation (CCG): due to the spatial displacement of the receptive fields of LGN 1 and LGN 2, shown in the inset on the left in *A*, the spiking response (black) was anti-correlated at the temporal frequency of the grating (orange). **(C)** Stimulus driven activity of the same LGN cells shown in **(A)** during the presentation of the natural stimulus. The neuronal activity is characterized by transient events of variable duration. Note that, in contrast to **(A)**, the events of LGN 1 and LGN 2 are now weakly but positively correlated. **(D)** Left, temporal auto-correlation: the timescale of the spiking response (gray = LGN 1, black = LGN 2) is much shorter than the timescale of the natural stimulus (orange). Right, temporal cross-correlation: on average LGN 1 and LGN 2 are weak but positively correlated due to the broad spatial correlations in the natural stimulus (Desbordes et al., 2008). The *in vivo* recordings in the LGN have been conducted in the group of Jose-Manuel Alonso.

We first tested LGN cells with drifting gratings, i.e., sinusoidal modulation of luminance along the motion axis orthogonal to the grating orientation. As illustrated in Figure 2A, LGN neurons were strongly driven by this moving grating (stimulus evoked firing rate “FR,” see Materials and Methods; FR_DG_ = 15.72 spikes/s, *n* = 18). The precision of the temporal response, as quantified by the temporal width of the auto-correlation of the spiking response (response timescale “RTS,” see Methods and Materials; RTS_DG_ = 81.94 ms, *n* = 18; Figure 2B, left) was roughly set by the temporal frequency of the drifting grating (TF = 2 Hz in this example) and the spiking reliability was low (reliability “REL,” see Materials and Methods; REL_DG_ = 0.025, *n* = 18). Because a grating is defined as a spatial periodic luminance stimulus pattern, simultaneously recorded LGN neurons having receptive fields of the same sign (ON or OFF) and separated by roughly half the grating's period (Figure 2 inset on the left) showed anti-correlation in their spiking responses (Figure 2A, compare LGN 1 and LGN 2; Figure 2B, right). Thus, a moving sinusoidal grating shapes both the temporal and spatial properties of LGN inputs onto cortical area V1: the grating temporal frequency imposes the timescale of LGN responses and its spatial frequency controls the spatial correlations within the LGN.

We then investigated the dynamics of the same LGN cells evoked by a complex, natural stimulus. The stimulus was taken from Baudot et al. (2013): a single full field, static natural image (see Figure 1C, left) is scanned with realistic cat eye-movements to emulate the retinal flow produced by the active exploration of a natural scene (see Materials and Methods). In general, natural stimuli are characterized by broad spatial and temporal correlations (Field, 1987), which are very different from the spatiotemporal correlations of the grating stimulus. In contrast to the periodic spiking responses seen with drifting gratings, spiking responses during natural stimuli exhibited complex temporal profiles as illustrated in Figure 2C. LGN neurons exhibited a diverse mixture of active periods and quiet epochs (FR_NI_ = 8.56 spikes/s, REL_NI_ = 0.026, *n* = 18), as was already previously observed using a “cat-cam” movie (Butts et al., 2007, 2010; Desbordes et al., 2008). This observation was also confirmed with the natural stimulus animation designed by Baudot et al. (2013) (Figure 2C) and used in the present study. Overall, the temporal precision of the LGN spiking activity was smaller than the temporal precision of the natural stimulus [RTS_NI_ = 17.49 ms, *n* = 18; Figure 2D, left: compare auto-correlation of the stimulus (orange) and spiking response (black, gray)]. This property highlights the temporal de-correlation and whitening of the power spectrum of the evoked responses observed in LGN cells during natural movies (Dan et al., 1996). However, the temporal de-correlation of the stimulus by LGN cells was not the only difference found between grating and natural stimulus conditions. We found that the same LGN cell pair that showed anti-correlated activity during drifting gratings (Figures 2A,B right) showed correlated spiking activity during natural stimuli (Figures 2C,D right). This can be understood by considering that the average spatial correlations in natural scenes are broad (Field, 1987) and induce correlations within the retina (Pitkow and Meister, 2012) and LGN (Desbordes et al., 2008), albeit with a reduced spatial extend (Pitkow and Meister, 2012).

In summary, the spatiotemporal correlations of LGN responses to either drifting gratings or natural scenes appear to be very different. While the response timescale of individual LGN cells are similar to the stimulus timescale during drifting gratings, LGN responses are temporally de-correlated during natural stimuli (Dan et al., 1996) and are characterized by a mixture of active and quiet epochs (Butts et al., 2007, 2010; Desbordes et al., 2008, 2010). Furthermore, the spatial stimulus profiles induced distinctive correlations among LGN cells during drifting gratings and natural stimuli, the later causing correlated firing whose strength decayed with receptive field distance (Desbordes et al., 2008). How this stimulus dependent LGN activity is processed by the push-pull receptive field organization of V1 simple cells and how short-term synaptic depression modulates the thalamic drive under these conditions is not known and will be investigated using a model of the thalamo-cortical visual system in the remaining part of this study.

### Realistic model of the thalamo-cortical visual pathway

**Figure 3:**
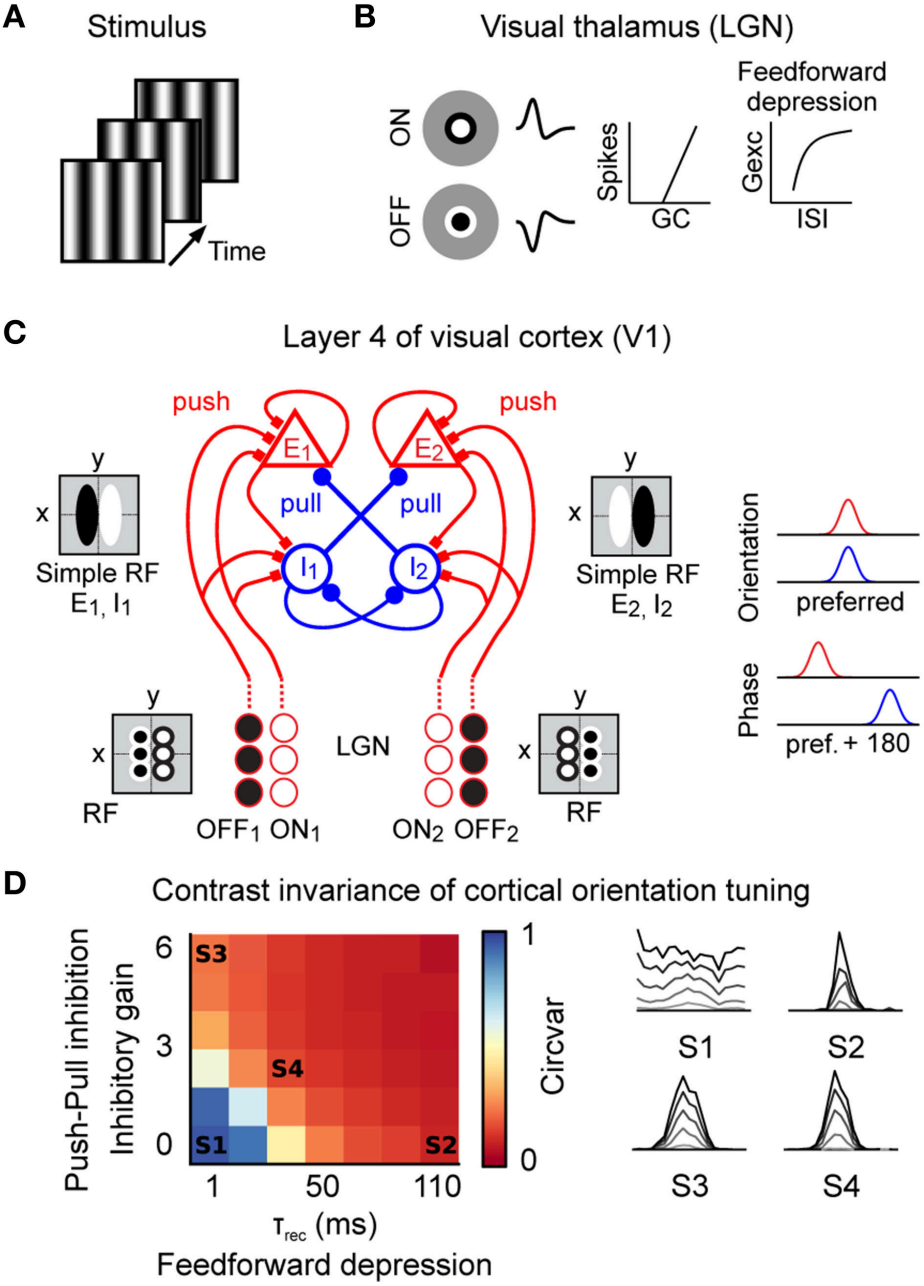
**Spiking model of the early visual system**. **(A)** The stimulus was modeled as a sequence of frames presented at 150 Hz (grating stimulus shown here). **(B)** Model of the visual thalamus (lateral geniculate nucleus “LGN”). ON and OFF center cell populations were modeled by linear spatiotemporal receptive fields followed by a non-linear spike generation mechanism. Thalamo-cortical synapses implemented short-term depression (feedforward depression). **(C)** Prototypic recurrent network model of layer 4 in the cat primary visual cortex “V1” with correlation-based connectivity implementing the push-pull receptive field organization. Inputs from the LGN provide direct excitatory (push). In cat V1, inhibitory neurons project preferentially to neurons having a receptive field phase difference of around 180°, effectively implementing the push-pull inhibition. Note also the intracortical reciprocal inhibition between inhibitory I1 and I2 neurons (Kayser and Miller, 2002) and the intracortical excitatory amplification for E1 and E2 neurons. Neurons were modeled as conductance based leaky-integrate-and-fire neurons. **(D)** Level of contrast invariant orientation tuning of the model in the complete parameter space of feedforward depression (τ_rec_) and push-pull inhibition (inhibitory gain). Orientation tuning curves of example parameter combinations (S1–S4) at different contrast values (gray = low contrast, black = high contrast). S1 = model without push-pull inhibition and feedforward depression; S2 = model with feedforward depression; S3 = model with push-pull inhibition; S4 = model with push-pull inhibition and feedforward depression.

To study the effect of the push-pull organization within the classical receptive field and synaptic depression at the thalamo-cortical synapse in sensory processing we implemented a conductance-based model of the thalamo-cortical visual system. As detailed above, our model was inspired by the classical modeling of push-pull receptive field organization of V1 simple cells developed by Troyer et al. (1998) (Figure 3). In addition to the parameters already present in the Troyer's model (Troyer et al., 1998), we introduced a minimal number of variables (e.g., inhibitory synaptic strength) that were calibrated to reproduce the response dynamics of V1 simple when presented with oriented gratings. Please note, more complex models of the thalamo-cortical processing in the visual system have been developed (e.g., Lauritzen and Miller, 2003). However, as our main aim was to investigate the role of push-pull receptive field organization and feedforward synaptic depression in sensory processing of natural stimuli we build upon the original Troyer's model as this model implements these circuit elements in a simple and comprehensible manner. Our rationale was to investigate under which parameter ranges such a simplified model could also contribute to the sparse and temporally precise encoding of natural stimuli. We are fully aware of the fact that not all simple cells in V1 show a push-pull receptive field organization (Martinez et al., 2005) and that a more complete model of cat V1 processing should include lateral connections to allow for center-surround interactions. However, we reasoned that even such a simple model will provide insights into the stimulus dependent sensory processing along the thalamo-cortical pathway.

The stimuli were modeled by a sequence of images (Figure 3A) updated at a refresh rate of 150 Hz. Spiking input to V1 was obtained by convolving the stimuli movies with the linear spatiotemporal receptive fields of two populations of ON-center and OFF-center geniculate neurons followed by a non-linear spiking mechanism (Figure 3B). Despite its simplicity, this LGN model indeed captured the essential stimulus-dependent spiking responses observed *in vivo* (Figure 2), e.g., responses of neighboring LGN neurons were anti-correlated during drifting grating conditions but correlated during natural stimuli (data not shown). Short-term plasticity at the thalamo-cortical synapse effectively implemented feedforward depression onto cortical cells (Banitt et al., 2007; Figure 3B). At the level of V1, we implemented a prototypical push-pull network of reciprocally connected excitatory and inhibitory cortical neurons in the thalamic input layers of higher mammals (Troyer et al., 1998; Figure 3C). In this framework, excitatory and inhibitory neurons were more likely to be connected to neurons with similar orientation preference (Figure 3C, right). Excitatory connections were established between neurons of similar phase but inhibitory neurons connected preferentially to cells having opposite phase. Such connectivity effectively mimics the push-pull receptive field organization of cortical simple cells (Palmer and Davis, 1981; Ferster, 1988; Tolhurst and Dean, 1990; Troyer et al., 1998). Ongoing background activity was modeled as independent Poisson processes (see Materials and Methods).

In order to constrain our model, we reasoned that it should reproduce, for a biologically plausible regime of the model's parameters, one of the most fundamental functional properties of excitatory V1 neurons, that is contrast-invariant orientation tuning (Sclar and Freeman, 1982; Troyer et al., 1998; Banitt et al., 2007). To achieve a full parametrical bi-dimensional exploration, we systematically varied the strength of the push-pull inhibition and the strength of the feedforward depression and estimated the orientation tuning at various contrast levels (Figure 3D, right). To illustrate how the orientation tuning was dependent on the strength of the push-pull inhibition and feedforward depression we calculated the circular variance (Ringach et al., 2002) of the responses to a high contrast grating (Figure 3D, left). A circular variance of 1 would indicate a very broad / no orientation tuning (e.g., Figure 3D, S1) wile a circular variance of 0 would indicate narrow orientation tuning (e.g., Figure 3D, S4). The strength of the push-pull inhibition was varied by the “inhibitory gain,” i.e., the ratio of the inhibitory and excitatory synaptic kernel area. The impact of the feedforward depression was regulated by the recovery time constant “τ_rec_” of the depressing synapses (τ_rec_ = 1–110 ms). In the absence of push-pull inhibition (inhibitory gain = 0) and feedforward depression (τ_rec_ = 1 ms), the model failed to implement pronounced contrast-invariant orientation tuning as made evident by the high value of the circular variance (Figure 3D, left - state S1) and the progressive loss of stimulus selectivity in the tuning curves at higher contrast values (Figure 3D, right - S1, note that the orientation preference from the converging LGN inputs is visible at low contrast only (gray line), see Materials and Methods). As already shown by others, increasing either feedforward depression (Figure 3D, S2) or push-pull inhibition (Figure 3D, S3) alone was sufficient to obtain an orientation tuning largely invariant to contrast changes (Troyer et al., 1998; Banitt et al., 2007). Please note that, due to the architecture of the Troyer model, cortical inhibitory neurons showed less pronounced orientation tuning (see Figure 8D in Troyer et al., 1998), which is in agreement with experimental data from putative inhibitory neurons in layer 4 of cat V1 (Cardin et al., 2007).

Since we focused on the contribution of cortical inhibition and thalamo-cortical adaptation on sensory processing we did not investigate the potential contribution of LGN variability (Sadagopan and Ferster, 2012) and synchrony (Kelly et al., 2014) onto the emergence of cortical feature selectivity. Under these assumptions, and once that the model had been calibrated on these basic response properties, we explored the stimulus-dependent evoked response dynamics illustrated in Figure 1. To do so, we selected realistic values for feedforward depression and push-pull inhibition (Troyer et al., 1998; Banitt et al., 2007) (τ_rec_ = 30 ms, inhibitory gain = 2, state S4 in Figure 3D) and studied the model's responses to better understand the role of push-pull receptive field organization and synaptic depression in sensory processing of artificial and natural stimuli.

### V1 responses during drifting gratings in the model

**Figure 4:**
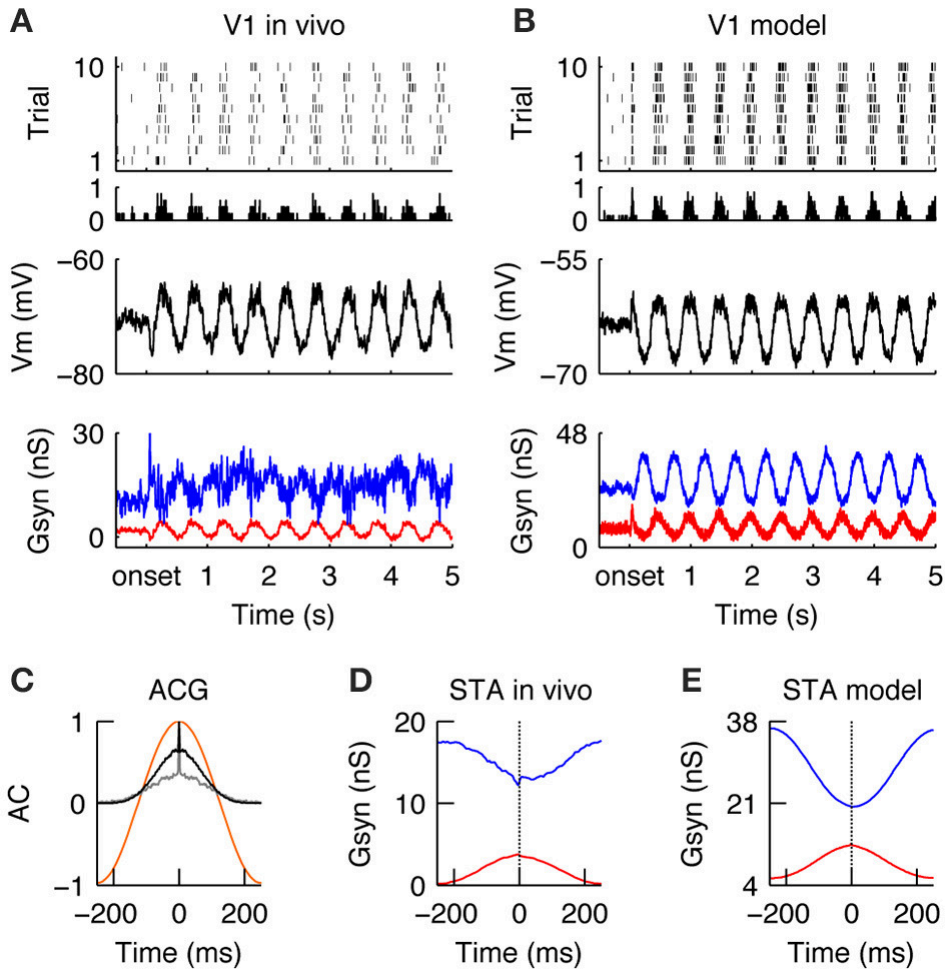
**V1 responses during drifting gratings. (A)** Spiking (top), membrane potential (Vm, middle) and synaptic conductances (Gsyn, bottom) responses of simple cell recorded intracellularly *in vivo* during the grating stimulus. G_*exc*_ (red) and G_*inh*_ (blue) synaptic input, averaged across trials. Note periodic dense spiking activity caused by anti-correlated G_*exc*_ and G_*inh*_. **(B)** Spiking and sub -threshold responses of a modeled simple cell during the presentation of a grating. Note the qualitative similarity to the *in vivo* condition. **(C)** Auto-correlation of the average spiking responses in the model (black) and *in vivo* (gray) of the cells shown in **(A,B)**. The temporal auto-correlation of the stimulus is shown in orange. **(D,E)** Spike-triggered average (STA) of G_*exc*_ and G_*inh*_ *in vivo* **(D)** and in the model **(E)**. The anti-phasic relationship between G_*exc*_ and G_*inh*_ creates a wide “spiking opportunity” window.

**Figure 5:**
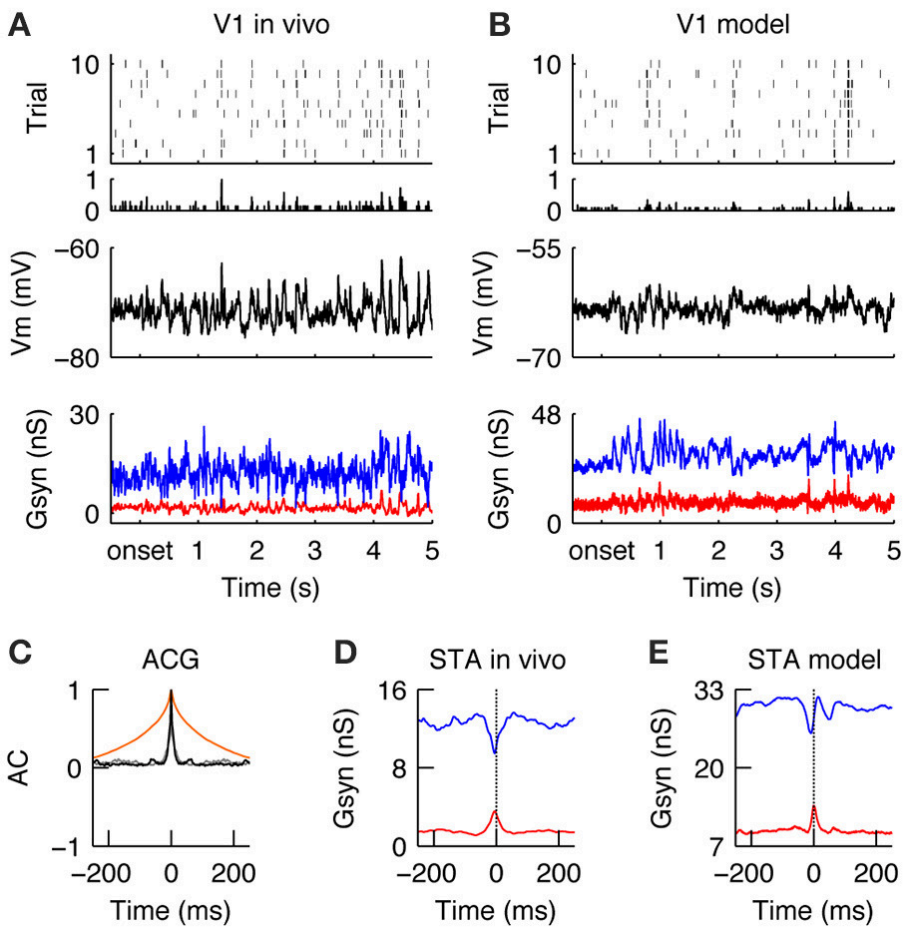
**Encoding of natural stimuli by V1 simple cells. (A–E)** Same format as Figure 4. **(A,B)** Spiking and sub threshold responses during the natural stimulus of the same simple cells *in vivo* **(A)** and in the model **(B)** as shown in Figure 4 during the grating stimulus. Note the low firing rates with reliable events caused by complex Vm waveforms and balanced G_exc_ and G_*inh*_. **(C)** The response timescales (gray = *in vivo*, black = model) are much shorter as compared to the temporal correlation in the natural stimulus (orange) and the responses during the grating stimulus (compare to Figure 4C). **(D,E)** The STA of G_*exc*_ and G_*inh*_ show that spikes are driven during natural conditions by a transient reduction of inhibition with a simultaneously transient increase of excitation. This transient unbalance of G_*exc*_/G_*inh*_ creates a tight “spiking opportunity” window that can explain the short response time constant during the natural stimulus **(C)**.

Similar to the LGN, during stimulation with drifting gratings, the spiking activity in V1 was modulated at the stimulus temporal frequency (TF = 2 Hz) (Figures 4A,B, for a qualitative comparison we present an *in vivo* example from the study Baudot et al. (2013) next to the modeling results). The spiking response was dense (FR_DG_ = 6.9 spikes/s, at the model configuration = τ_rec_ = 30 ms, inhibitory gain = 2. Please see below and Figure 5 for the model responses of the τ_rec_/inhibitory gain state-space) and the temporal precision was broad (RTS_DG_ = 52.52 ms, Figure 4C). The membrane potential showed a typical push-pull behavior (Figures 4A,B Vm), due to counter-phase synaptic G_exc_ and G_*inh*_ waveforms (Figures 4A,B, G_*syn*_)—a well-known property of simple cells in cat V1 in response to an optimal drifting grating (Anderson et al., 2000; Monier et al., 2003; Priebe and Ferster, 2005; Tan et al., 2011; Baudot et al., 2013). These counter-phase waveforms are expected from the spatiotemporal correlations of the LGN neurons (Figure 2) and the push-pull receptive field organization of the V1 simple cell (Figure 3C; Troyer et al., 1998). By consequence, spike generation was driven by small G_exc_ fluctuations, riding on a slower component (Figures 4D,E), resulting in low response spiking reliability (REL_*DG*_ = 0.023). In addition to the counter-phase component, inhibition also showed a DC offset (Figures 4A,B)—likely originating from stimulus independent recurrent processing and/or complex inhibition (Lauritzen and Miller, 2003). In contrast to this un-balancing during preferred orientation, when a drifting grating of non-optimal orientation was displayed, G_*exc*_ and G_*inh*_ became temporally overlapping and balanced (Troyer et al., 1998; Monier et al., 2008), leading to shunting effects (Borg-Graham et al., 1998; Monier et al., 2003, 2008). Consequently, the membrane potential remains below the spiking threshold most of the time, and only a few spikes are elicited in this configuration (data not shown).

In summary, V1 simple cells in the model and *in vivo* responded to drifting gratings of optimal orientation by slowly varying G_*exc*_/G_*inh*_ waveforms of opposite phase, causing G_*exc*_ and G_*inh*_ to be unbalanced. Consequently, during the periods of evoked excitation, the simulated and recorded spike responses were dense, variable, and temporally imprecise and in general unconstrained by the out-of-phase evoked inhibition (Figures 4D,E).

### Sparse and precise encoding of natural stimuli by V1 simple cells

Once the neuronal responses to drifting gratings had been characterized, our next objective was to study how the same model would respond to natural stimuli. Once again, the *in vivo* responses were taken from a previous publication (Baudot et al., 2013) and used here in the purpose of illustrating the contextual reshaping of evoked sub-threshold and spiking responses. A more detailed analysis of this *in vivo* data was given in Baudot et al. (2013). Overall, the global V1 spiking activity was reduced ~3-fold as compared to grating stimulus (FR_*NI*_ = 2.38 spikes/s, Figures 5A,B; compare to Figure 4). The membrane potential showed a complex profile (Figures 5A,B, middle) since G_*exc*_/G_*inh*_ synaptic inputs were now balanced and tightly correlated (Figures 5A,B, bottom, Figures 5D,E, STA). This is in strong contrast with the unbalanced G_*exc*_/G_*inh*_ ratio obtained with gratings at optimal orientation, as can be seen by directly comparing Figures 4, 5. The fine temporal relationship existing between G_*exc*_ and G_inh_ varied over time, with G_*exc*_ and G_*inh*_ being unbalanced only occasionally, resulting in a spiking response whose strength and temporal precision are dependent on both, the amount of input synchrony and the temporal width of the G_*exc*_-G_*inh*_ anti-correlation. Therefore, on average, spikes were driven by transient anti-correlations between G_*exc*_ and G_*inh*_ (i.e., large G_*exc*_ peaks and/or drops in G_*inh*_; Figures 5D,E), resulting in transient spiking events across trials (RTS_*NI*_ = 19.07 ms, REL_*NI*_ = 0.026 Figure 5C).

We further investigated the reasons of such transient imbalances between G_*exc*_/G_*inh*_ with natural images. We found that they were caused by the interplay between the broad spatial correlation of the stimuli and the push-pull structure of the classical V1 receptive field. When the push-pull receptive field was covered by either a non-optimally oriented local contrast or an homogenous luminance patch, the V1 neuron received a simultaneous synaptic “push” (G_*exc*_) and “pull” (G_*inh*_), which caused a shunt in the excitatory drive (Borg-Graham et al., 1998). Only when a local contrast at the preferred orientation hits the V1 neuron's receptive field, then this tight balance of G_*exc*_/G_*inh*_ is briefly released in favor of excitation. Moreover, rapid changes in the luminance pattern sometimes trigger transient, highly synchronous LGN inputs, which pass through this short “spiking opportunity window” of the V1 neuron and thus successfully generate action potentials (Kremkow et al., 2010a,b). However, most of the time, the tight balance and correlation between G_*exc*_ and G_*inh*_, inducing a short integration window that filters out non-inputs (Kremkow et al., 2010a,b) and hence the cell remained silent.

This cell behavior is in sharp contrast to that observed with the drifting grating stimulus. Since both grating spatial frequency and orientation were optimized to fit the V1 neurons' receptive fields, the push-pull mechanism is alternated in time and G_*exc*_ and G_*inh*_ are anti-correlated. Due to the low temporal frequency of the grating, long epochs of strong conductance imbalance alternately favoring G_*exc*_ and G_*inh*_ are observed, resulting in dense firing during periods of reduced inhibition (Figure 4). Consistent with this view, increasing the temporal frequency range by modulating a static grating image with the same pattern of eye movements or by presenting a dense noise stimulus resulted in a faster dynamics of the excitation and inhibition, both *in vivo* (Baudot et al., 2013) and in the model (see Figures S1, S2). In both cases (recorded and simulated), the spiking responses to grating and eye movements (GEM condition) were more reliable as compared to the responses to dense noise (DN), likely because the spatial frequency and orientation of the grating were matched to the V1 neurons' receptive fields, resulting in a denser response level. This difference was not apparent at the subthreshold level. The interpretation given in Baudot et al was that spiking reliability in DN conditions compared to GEM was lessened by the reduction in low-frequency power in the subthreshold activity, leading to larger trial-to-trial variability in the trespassing of spike activation threshold.

In summary, when stimulated with a natural stimulus, V1 simple cells respond with low firing rates but in a temporally transient manner (Figures 5A,B). Such behavior is due to the dynamic regime of G_*exc*_ and G_*inh*_ caused by the broad spatial and temporal correlations of natural images and its interaction with the push-pull receptive field organization. Most of the time, G_*exc*_ and G_*inh*_ were tightly balanced, the spiking output was sparse and spikes were only elicited during epochs of transient unbalanced G_*exc*_/G_*inh*_.

### Interplay between push-pull inhibition and feedforward depression during natural stimuli

We have shown above that a thalamo-cortical model with push-pull inhibition and feedforward depression can simulate realistic V1 responses to both drifting gratings and natural stimulus stimulations. The model uses a single set of biologically realistic parameters and examples shown in Figures 4, 5 were obtained with τ_rec_ = 30 ms and an inhibitory gain of 2. Since a detailed, quantitative examination of push-pull inhibition and feedforward depression strengths *in vivo* is still yet unavailable, we took advantage of our parametric model to explore the sensitivity of cortical dynamics to different strengths of push-pull inhibition and feedforward depression. Our objective was to define the most appropriate parametric region where spiking responses and intracellular membrane potential trajectories showed realistic stimulus-dependent V1 response properties. In particular, we were interested in investigating the extent in which each mechanism alone could account for the sparse and precise responses during natural stimuli, or whether both needed to be recruited simultaneously. To address this question, we studied the complete push-pull inhibition and feedforward depression space by varying systematically the inhibitory gain (push-pull inhibition) and recovery time constant (τ_rec_) of feedforward depression (Banitt et al., 2007)

A small τ_rec_ would result in a fast recovery and, thus, in a weak depression, whereas a large τ_rec_ would produce both slow recovery and strong depression. There are other factors that change the amount of synaptic depression (e.g., the rate of depression), however, we chose to vary τ_rec_ because it allows us to control the timescale of depression. The minimal τ_rec_ was set to 1 ms, that is, smaller than the refractory period of LGN neurons and, hence, resulting in no depression. The maximally allowed τ_rec_ was set within the physiological range (τ_rec_ = 110 ms) (Banitt et al., 2007). The push-pull inhibition strength was varied by changing the strength of the inhibitory synapses in the V1 network, resulting in a graded control of the balance between excitation and inhibition. Note that an inhibitory gain of zero would result in pure feedforward excitation, whereas a large one, as scaled to the maximal admissible value, would result in strong push-pull inhibition.

**Figure 6:**
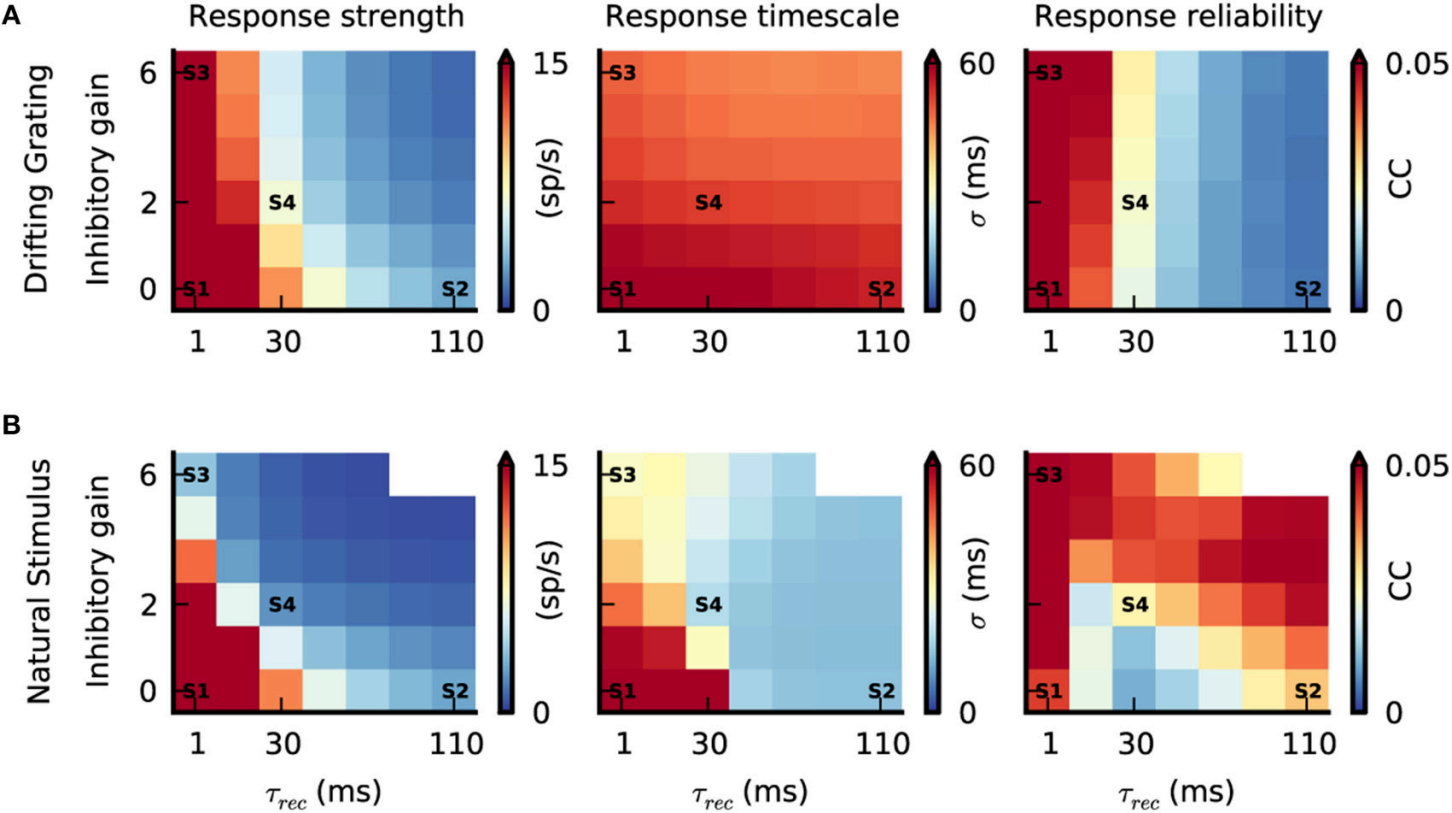
**Interplay between push-pull inhibition and feedforward depression. (A,B)** Response strength, response timescale and response reliability of simulated V1 simple cells as a function of feedforward depression (τ_rec_) and push-pull inhibition (inhibitory gain). The labels S1–S4 are the parameter configurations shown in Figure 3D. **(A)** Response characteristics during grating stimulation. While increasing τ_rec_ reduces response strength and reliability varying the inhibitory gain has no prominent effect due to the anti-phasic relationship of G_exc_ and G_inh_ during the grating stimulus (Figure 4). The response timescale is large and similar across the parameter space. **(B)** Response characteristics during the natural stimulus. Due to the correlated G_exc∕_G_inh_ during natural stimuli (Figure 5), increasing τ_rec_ and inhibitory gain reduces the response strength and response timescale. The response reliability changes as a function of both parameters, and can even reach high values at low response strength (combine bottom top and right panels in Figure 6B).

To compare these different parametric regimes, we quantified the activity of the model by estimating the stimulus evoke firing rates, response timing precision and response reliability. Figure 6 shows these measurements at all positions of the state space and for both, the grating and natural stimulus. Below, we will discuss the effect of systematically varying the strength of the push-pull inhibition and feedforward depression in the model.

Inactivating both push-pull inhibition (inhibitory gain = 0) and feedforward depression (τ_rec_ = 1 ms) resulted in strong V1 responses for both stimuli (Figures 6A,B, “Response strength,” state S1) and with such high firing rates, the reliability was always high (Figures 6A,B, “Response reliability”, state S1). By contrast, temporal precision —and by consequence response timescale— were clearly stimulus dependent (Figures 6A,B, “Response timescale”). Response timescale was large for both grating and natural stimuli. This can be understood when considering the spatiotemporal properties of the different stimuli. The grating temporal frequency sets the response timescale (Figure 6A, state S1) (Troyer et al., 1998) while, during stimulation with natural images, the long temporal correlations cause correlated V1 responses and hence a long response timescale (Figure 6B, state S1). This first result demonstrates that the temporal integration of excitatory thalamic inputs *per se* is not sufficient to elicit sparse, precise V1 responses during natural stimuli in our model.

Increasing τ_rec_, while keeping push-pull inhibition inactivated, resulted in a decrease of V1 response amplitudes (Figure 6, state S2) because the LGN synaptic drive was attenuated by the depressing synapses. Note however, that the sparsening of the responses observed during the natural stimulus animation was accompanied by a decrease in the response reliability for intermediate values of τ_rec_ (Figure 6B, τ_rec_ = 30 ms), and by an increase in response reliability for large values (Figure 6B, state S2). For drifting gratings the response reliability was monotonically decreasing (Figure 6A, S2). For both stimuli, the response timescale decreased, however, the reduction was much more prominent during the natural stimulus (Figures 6A,B, state S2). This difference is explained by the temporal filtering properties of depressing synapses that emphasize transient inputs (Chance et al., 1998) similar to those evoked by abrupt changes in the natural stimulus. Note that transient and temporally precise cortical responses caused by feedforward depression filtering were most evident when the cortical neurons were in a quiet state (i.e., low level of ongoing activity). With drifting gratings, the response timescale was larger and not affected by the change in τ_rec_ because the temporal changes were slower than the depression with the longest time constant and thus response reliability decreased.

Increasing the strength of the push-pull inhibition, while keeping the feedforward depression inactivated (τ_rec_ = 1 ms), had no significant effect on V1 spiking responses during the grating stimulus (Figure 6A, state S3). This is because G_exc_/G_inh_ are unbalanced during these stimuli. In contrast, increasing the inhibitory gain lowered both response strength and response timescale during the natural condition (Figure 6B, state S3), as expected from the tight G_exc_/G_inh_ balance under such stimulation (Figure 4). At the same time, response reliability remained high, despite the low firing rate. This is due to the selective filtering of transient inputs by the push-pull inhibition, equivalent functionally in recurrent networks to the feedforward inhibition mechanism modeled by Kremkow *et al* in feedforward networks (Kremkow et al., 2010a,b). Note that in state S3, push-pull inhibition alone was sufficient to obtain contrast-invariant orientation tuning (Figure 3D; Troyer et al., 1998). However, for this particular state, the absolute level of G_inh_ needed to balance the impinging G_exc_ to obtain both sparse and reliable V1 responses reached values that were not biological-plausible.

As demonstrated with state S4, our realistic conditions corresponding to both weak feedforward depression (τ_rec_ = 30 ms) and moderate push-pull inhibition (inhibitory gain = 2) is a subpart of the parametric space where model V1 spiking responses (repeated across trials) remarkably reproduce those observed *in vivo* (Figure 6B, state S4 and Figure 5). The absolute G_inh_ values remained in a biologically realistic regime (Figure 5B) and spikes were elicited by strong and transient G_exc_ inputs with simultaneous withdrawal of G_inh_ (see model STA Figure 5E). Interestingly, for strong push-pull inhibition (inhibitory gain > 2) and long feedforward time constants (τ_rec_ > 30 ms), response reliability during stimulation with natural images was even higher than found with optimal state S4. Such high reliability came at the cost of the neuronal responsiveness, as shown by the very low response strength in this region of parameter space (Figure 6B).

In summary, our comprehensive parametric space analysis demonstrates that both push-pull inhibition and feedforward depression, each by itself, can reduce response amplitude in V1, while increasing temporal precision to achieve a sparse and temporally precise cortical representation of natural stimuli. However, there is a small range of paired values for which their interplay results in realistic dynamics, switching from dense/unreliable to sparse/reliable when increasing stimulus complexity.

## Discussion

The objective of our study was to demonstrate the respective impact of push-pull inhibition and feedforward depression in evoked cortical dynamics, at the level of the first-order simple cells in visual cortex. To isolate their contribution, we deliberately did not take in consideration the possible role of intralaminar, intracortical lateral and feedback connectivity. Rather, recurrent connectivity was limited in our model to its simplest expression, which is the push-pull organization well established for cortical cells receiving a direct thalamic input in cat and monkey primary visual cortex (Troyer et al., 1998; Miller et al., 2001). Still, such a simple model can explain several aspects of cortical dynamics to natural scenes.

### Functional equivalence between feedforward and push-pull inhibition

Both balance and temporal interplay between excitation and inhibition are key factors in determining spiking responses [e.g., Figure 8 in (Monier et al., 2008) and (Gerstein and Mandelbrot, 1964; Wehr and Zador, 2003; Kumar et al., 2008b; Okun and Lampl, 2008; Vogels and Abbott, 2009; Kremkow et al., 2010a,b; Graupner and Reyes, 2013)]. The feed-forward inhibition motif posits that a thalamic neuron connects to the same excitatory cortical cell through both a monosynaptic feed-forward excitatory connection and a di-synaptic relay recruiting an inhibitory cortical cell. This connectivity pattern has been demonstrated in both somatosensory (Swadlow, 2003; Cruikshank et al., 2007) and auditory primary cortices (Wehr and Zador, 2003) and imposes a temporal lag of few milliseconds between excitatory and inhibitory inputs converging onto the same target neuron. This very short delay is biophysically related to conduction time, synaptic delay transmission and time-to-reach threshold. By consequences, the interplay between “early” excitation and “delayed” inhibition opens an highly selective “opportunity window” filtering out temporally uncorrelated inputs (Isaacson and Scanziani, 2011) and allows reliable and efficient transmission of information in noisy environments (Kremkow et al., 2010b).

Although a recent study based on V1 extracellular recordings has shown that adaptation to fast contrast changes similar to natural stimuli, can be accounted for by an equivalent feedforward inhibitory circuit where dominant inhibition lags dominant excitation by 10 ms (Levy et al., 2013), structural evidence for such connectivity has not been found in V1 of higher mammals (cats and monkeys). Note here that the timing delays between excitation and inhibition simulated by a feedforward inhibition-like model in V1 are one order of magnitude larger (10 ms) than those reported above for both S1 and A1 (1 ms). This is because, unlike feedforward inhibition, push-pull inhibition relies on local recurrent connectivity and originates from inhibitory receptive fields of opposite phase compared to the target neuron (Miller et al., 2001). In spite of this structural specificity of the visual system that may distinguish higher mammals from rodents (reviewed in Fregnac and Bathellier, 2015), our model shows that push-pull inhibition becomes functionally equivalent to feedforward inhibition for input statistics that are not “optimal” for the push-pull structure, and results in an apparent asynchrony between excitation and inhibition. Strikingly, in rodents, drifting gratings elicit balanced excitation and inhibition in V1 neurons, which is in contrast to the push-pull behavior in cats (Figure 7 in Tan et al., 2011). It might be speculated that the push-pull receptive field circuitry of layer 4 neurons in V1 of higher mammals is an adaptation to the high visual acuity and/or related to the columnar organization of spatial phase in cat V1 (Wang et al., 2015). Overall, the message is that the relative timing between excitation and inhibition play a critical role in shaping transient responses of early sensory stages (Zhang et al., 2003; Wilent and Contreras, 2005; Kremkow et al., 2010a,b; Bruno, 2011), regardless of the underlying detailed connectivity patterns.

### A simple model for cortical dynamics with natural scenes

Our model provides novel insights into the role of the push-pull receptive field organization and feedforward depression in sensory processing of natural stimuli. It can reproduce several cardinal features of V1 simple cells, in particular a low stimulus-evoked firing rates together with both high spiking reliability and temporal precision during natural stimuli. These properties are caused by both a transient decrease of inhibition and an increase of excitation before spike onset (Haider et al., 2010). The strength of our model is its ability to characterize the optimal ranges of feedforward depression and push-pull inhibition values that are needed for eliciting such dynamics. Such a search cannot be done empirically *in vivo*. Increasing feedforward depression results in a general response strength reduction and when properly calibrated, feedforward depression emphasizes transient stimulus changes compared to sustained epochs. Similarly, push-pull inhibition results in a strong response strength reduction for the natural stimulus. Here excitation and inhibition become correlated because of the broad spatial correlations within the cortical receptive field. A contrast of proper orientation, spatial frequency and phase only occasionally covers the receptive field, causing excitation and inhibition to escape from the balanced regime (Figure 5). In contrast, when a grating stimulus matches the receptive field, excitation and inhibition remain out of phase most of the time (Figure 4).

Interestingly, it is the combination of feedforward depression and push-pull inhibition that proves to be beneficial. Although each process can implement orientation tuning on its own (Figure 3D; Troyer et al., 1998; Banitt et al., 2007), none alone reproduces sparse precise spiking, except for unrealistic inhibitory conductance level (push-pull inhibition) or abnormally low ongoing activity (feedforward depression). Thus, the push-pull receptive field organization endowed with feedforward depression leads to correlated excitation and inhibition during natural vision in simple cells. Remarkably, learning models have shown that simple receptive fields emerge when the network is trained to be sparse for natural images (Olshausen and Field, 1996). This suggests a fundamental link between the precision of the neuronal code, the push-pull architecture and the correlation between excitation and inhibition.

### Future extensions of the model

This study focuses on V1 cortical cells receiving direct thalamic afferents. Our model was rather schematic in relation to the organization specificity of layer 4 in mammals. Thus, it was not designed to reproduce the large biological diversity of receptive field structures and input conductance regimes that we, and others have previously reported intracellularly (Anderson et al., 2000; Monier et al., 2003, 2008; Priebe and Ferster, 2005; Cardin et al., 2010; Haider et al., 2010; Baudot et al., 2013). Note also that the spatial segregation of excitation and inhibition conductances in V1 layer 4 is found only in higher mammals (cats, monkeys) whereas it is absent in the rodents (Tan et al., 2011; Li et al., 2012, 2013, 2015b), a computational architecture difference reviewed in Fregnac and Bathellier (2015). These observations highlight the need to explore the contribution of other non-linear sub-thresholds properties of simple and complex cells and their adaptability as a function of input statistics (Fournier et al., 2011, 2014). In particular it will be of great interest to investigate how thalamo-recipient neurons in the rodent visual system, which have a very different receptive field organization as compared to higher mammals (reviewed in Fregnac and Bathellier, 2015), adapt to the statistics of the visual stimulus. The stimulus set used in Baudot et al. (2013) (drifting gratings, grating and eye movements, natural image and eye movements and dense noise) covers a wide range of input statistics with optimized (gratings) or non-optimized (natural scenes and dense noise) features and is therefore an ideal framework to investigate these questions in the future, both *in vivo* and in models (rodents and higher mammals).

Furthermore, several studies have demonstrated that center-surround mechanisms shape sparse and reliable responses to natural scenes (Vinje and Gallant, 2000, 2002; Guo et al., 2005). Our V1 model corresponds to a local cortical network in layer 4 of V1 lacking a “silent” surround. Future work will extend it to identify the impact of intra-laminar, long-range intra-cortical horizontal connections and feedback from higher cortical area (Bardy et al., 2006) on the temporal dynamics of V1 responses to natural inputs.

Future improvements of our conductance-based model will investigate several important aspects of naturalistic encoding in primary visual cortex. Ongoing activity should not be simplified to an unstructured “noise”: delayed correlations have been reported between excitatory and inhibition conductances in connected neurons in the ongoing state (Okun and Lampl, 2008). This “colored” noise shares similarities with cortical dynamics by natural scenes (see fractal analysis in El Boustani et al., 2009), and by interacting with the stimulus drive, it may impact on the precision of the code, as suggested by recent study in retinal ganglion cells (Cafaro and Rieke, 2010). Furthermore, it was shown that the behavioral state has a profound impact on ongoing and evoked activity in the visual system (Cano et al., 2006; Stoelzel et al., 2009; Niell and Stryker, 2010; Bereshpolova et al., 2011; Erisken et al., 2014; Zhuang et al., 2014; Schölvinck et al., 2015). Our model cannot reproduce these observations since the background input was modeled as stimulus-independent Poisson excitatory and inhibitory processes. Finally, one of our main findings in our intracellular recordings of V1 cells is that the stimulus-locked response variability *in vivo* at the sub-threshold level depends on the global (full field) context of the stimulation and is minimized for natural scenes when compared with other input statistics (Fregnac et al., 2005; Marre et al., 2005; Baudot et al., 2013), and on the amount of synaptic shunting due to an increase in inhibitory conductance (Monier et al., 2003). This suggests that the cortical dynamics cannot be simulated by stochastic models, but are compatible with near-the edge of deterministic chaos attractors when stimulus dimensionality becomes similar to natural scenes (Marre et al., 2009).

## Conclusion

Our original motivation was to explore the contribution of push-pull receptive field organization and feedforward depression on V1 responses to low dimension synthetic vs. naturalistic stimuli. It is often assumed that these spiking contrasted behaviors result from differences in input statistics (Carandini et al., 2005). More recent studies based on both spike and sub-threshold activities interpret the same observation as further evidence for stimulus-dependent intracortical adaptation (Fournier et al., 2011). Answers depend largely on the effectiveness of linear-non-linear receptive models to account for the full cortical multi-scale dynamics, from conductance to spike generation. First-order cortical cells, which receive a direct input from the thalamus, are considered to be the most linear neurons in the primary visual cortex. Other cells are considered to be more non-linear as they receive a considerable amount of long-range horizontal inputs (Stepanyants et al., 2009). Our modeling study helps clarifying the controversy, at least for first-order cortical cells, and indicates that the precision of the code and the sparsening of responses during natural stimulus may be taken as an evidence for the effectiveness of well-known non-linear interactions, push-pull receptive field organization and synaptic depression, in shaping the dynamics of cortical responses to high complexity scenes.

The simplicity of the mechanisms implemented here suggests that our results could be transposed to other sensory modalities and explored in other primary sensory cortices where some evidence pleads for an optimization of spiking reliability with natural statistics (Hromádka et al., 2008). They therefore provide a generic framework for a mechanistic approach of the interplay between excitation and inhibition in sensory processing. Our step-by-step parametric modeling approach demonstrates how sensory cortices process a broad range of stimulation statistics by tuning the balance and effective delay of excitation and inhibition (hence, sparseness) and the reliability of synaptic inputs (hence, temporal precision). This simplified architecture skeleton opens the door to incrementing future studies introducing other key elements such as recurrent/lateral interactions within the classical receptive field and beyond.

## Author contributions

JK, LP, CM, JA, YF, AA, GM designed research. JK implemented the model with input from LP. JK and JA conducted the *in vivo* LGN recordings and CM and YF the *in vivo* V1 recordings. JK, LP, CM analyzed data. JK, LP, CM, JA, YF, AA, GM wrote the manuscript.

### Conflict of interest statement

The authors declare that the research was conducted in the absence of any commercial or financial relationships that could be construed as a potential conflict of interest.

## Supplementary material

Figure S1 **V1 responses to a grating stimulus with simulated eye-movements. (A–E)** Same format as Figures 4, 5. **(A,B)** Spiking and sub threshold responses during the grating + eye-movement stimulus of the same simple cells *in vivo* **(A)** and in the model **(B)** as shown in Figures 4, 5 during the grating and natural stimulus, respectively. Note, the evoked responses are dense but, in contrast to the moving grating, temporally precise. **(C)** The response timescales (gray = *in vivo*, black = model) are similar as compared to the temporal correlation in stimulus (orange). **(D,E)** STA of G_exc_ and G_inh_ show that spikes are driven by a transient reduction of inhibition with a simultaneously transient increase of excitation, similar to the conditions during natural stimuli.

Figure S2 **V1 responses to a dense noise stimulus. (A–E)** Same format as Figures 4, 5. **(A,B)** Spiking and sub threshold responses during the dense noise stimulus *in vivo* **(A)** and in the model **(B)**. Note that dense noise evokes both temporally precise (peaks in the raster) and imprecise events (locked to the onset of the stimulus sequence). **(C)** Response timescales, gray = *in vivo*, black = model, orange = stimulus. **(D,E)** STA of Gexc and Ginh show that spikes are driven by a transient reduction of inhibition with a simultaneously transient increase of excitation.
