# Supplementary figures and images for: Push-Pull Receptive Field Organization and Synaptic Depression: Mechanisms for Reliably Encoding Naturalistic Stimuli in V1
Source: Front Neural Circuits. 2016 May 11;10:37. doi: 10.3389/fncir.2016.00037 (PMC4862982; doi:10.3389/fncir.2016.00037)

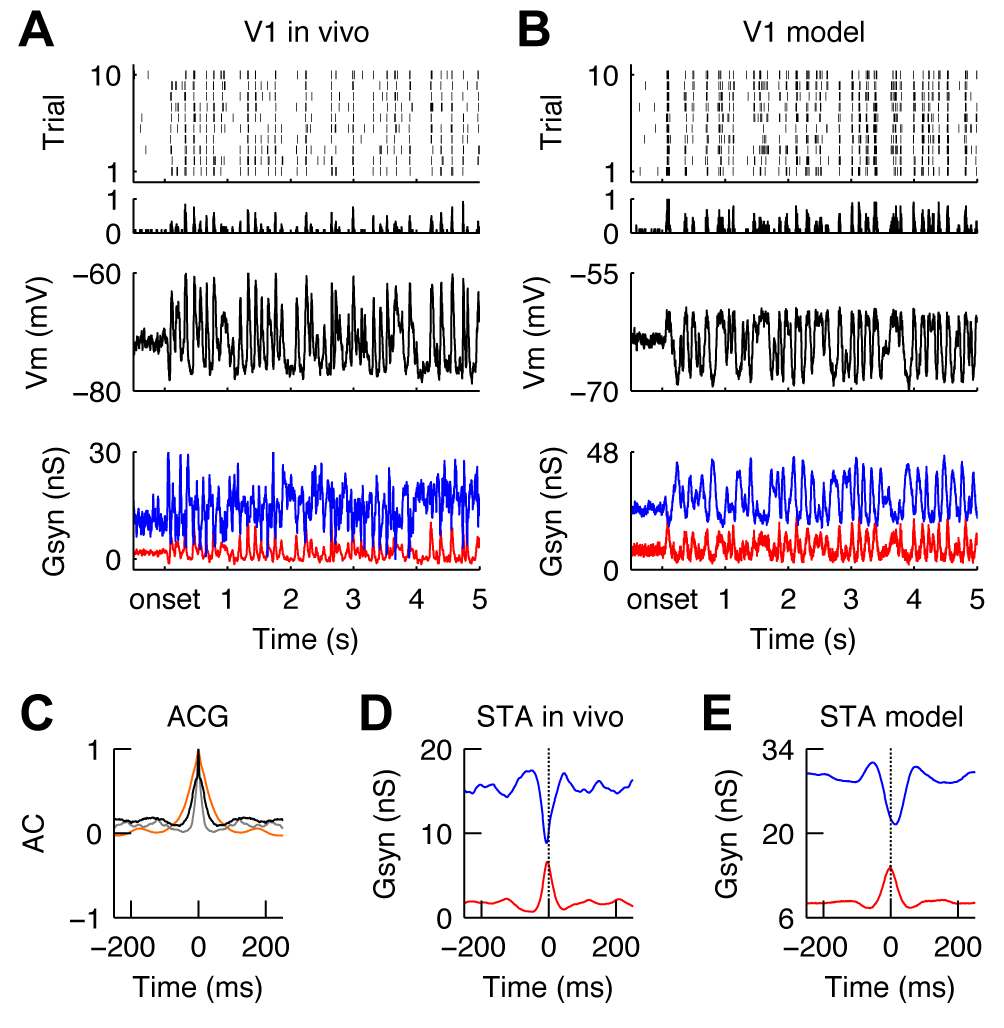

Supplement: Figure S1 — V1 responses to a grating stimulus with simulated eye-movements. (A–E) Same format as Figures 4, 5. (A,B) Spiking and sub threshold responses during the grating + eye-movement stimulus of the same simple cells in vivo (A) and in the model (B) as shown in Figures 4, 5 during the grating and natural stimulus, respectively. Note, the evoked responses are dense but, in contrast to the moving grating, temporally precise. (C) The response timescales (gray = in vivo, black = model) are similar as compared to the temporal correlation in stimulus (orange). (D,E) STA of Gexc and Ginh show that spikes are driven by a transient reduction of inhibition with a simultaneously transient increase of excitation, similar to the conditions during natural stimuli. [file Image1.tif]

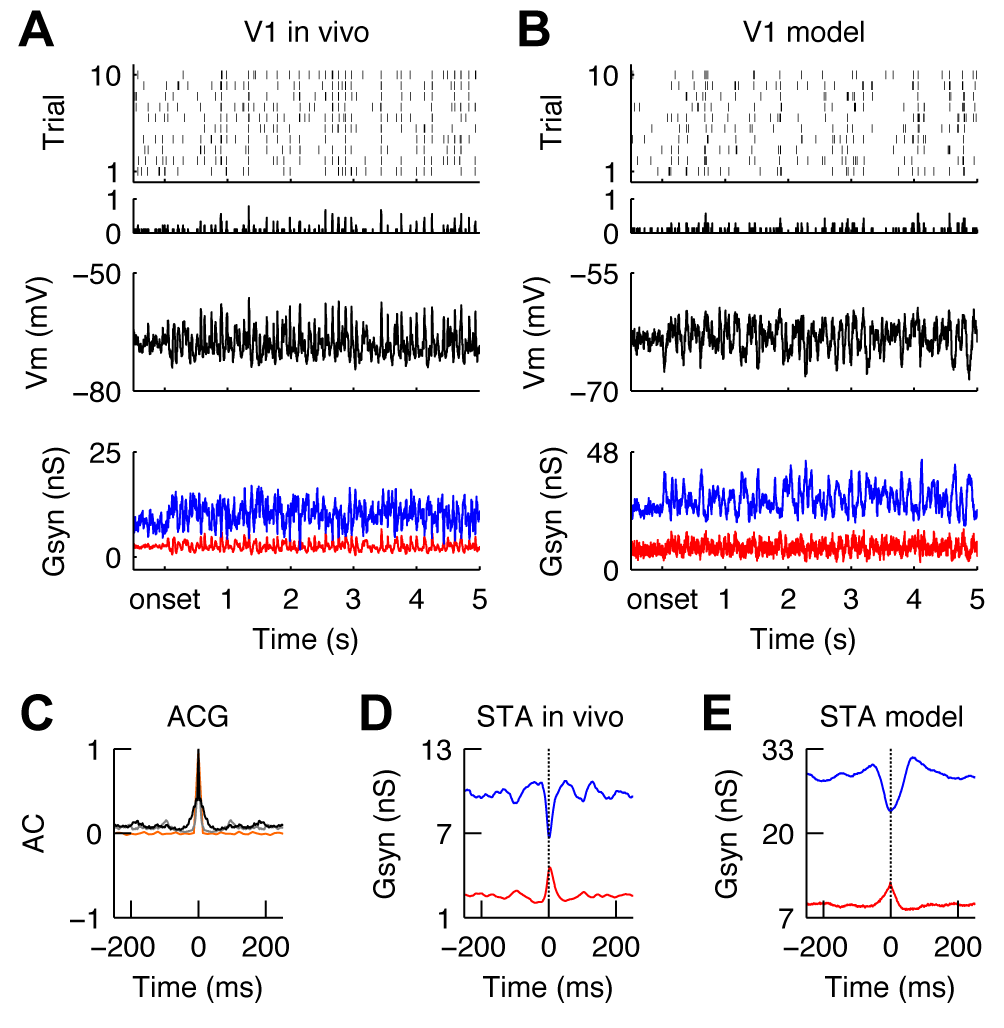

Supplement: Figure S2 — V1 responses to a dense noise stimulus. (A–E) Same format as Figures 4, 5. (A,B) Spiking and sub threshold responses during the dense noise stimulus in vivo (A) and in the model (B). Note that dense noise evokes both temporally precise (peaks in the raster) and imprecise events (locked to the onset of the stimulus sequence). (C) Response timescales, gray = in vivo, black = model, orange = stimulus. (D,E) STA of Gexc and Ginh show that spikes are driven by a transient reduction of inhibition with a simultaneously transient increase of excitation. [file Image2.tif]
